# Publisher Correction: Neddylation inhibition induces glutamine uptake and metabolism by targeting CRL3^SPOP^ E3 ligase in cancer cells

**DOI:** 10.1038/s41467-022-31203-9

**Published:** 2022-06-14

**Authors:** Qiyin Zhou, Wenyu Lin, Chaoqun Wang, Fei Sun, Siwei Ju, Qian Chen, Yi Wang, Yongxia Chen, Haomin Li, Linbo Wang, Zeping Hu, Hongchuan Jin, Xian Wang, Yi Sun

**Affiliations:** 1 Cancer Institute, the Second Affiliated Hospital, Zhejiang University School of Medicine, Hangzhou, 310009 China; 2 Key Lab of Biotherapy in Zhejiang, Sir Run Run Shaw Hospital, Zhejiang University School of Medicine, Hangzhou, 310016 China; 3 Department of Medical Oncology, Sir Run Run Shaw Hospital, Zhejiang University School of Medicine, Hangzhou, 310016 China; 4 Institute of Translational Medicine, Zhejiang University School of Medicine, Hangzhou, 310029 China; 5 Cancer Center, Zhejiang University, Hangzhou, 310058 China; 6 Department of Pathology, Affiliated Dongyang Hospital of Wenzhou Medical University, Dongyang, 322100 China; 7 School of Pharmaceutical Sciences, Tsinghua-Peking Joint Center for Life Sciences, Beijing Frontier Research Center for Biological Structure, Tsinghua University, Beijing, 100084 China; 8 Department of Surgical Oncology, Sir Run Run Shaw Hospital, Zhejiang University, Hangzhou, 310016 China; 9 Children’s Hospital, Zhejiang University School of Medicine, National Clinical Research Center for Child Health, Hangzhou, 310052 China; 10 Research Center for Life Science and Human Health, Binjiang Institute of Zhejiang University, Hangzhou, 310053 China

**Keywords:** Cancer metabolism, Neddylation, Ubiquitylation, Breast cancer

Correction to: *Nature Communications* 10.1038/s41467-022-30559-2, published online 31 May 2022.

The wrong Supplementary File was originally published with this article; it has now been replaced with the correct. The original article has been corrected.

## Supplementary information

Updated Supplementary Information

