# Supplementary material for: Neddylation inhibition induces glutamine uptake and metabolism by targeting CRL3SPOP E3 ligase in cancer cells
Source: Nat Commun. 2022 May 31;13:3034. doi: 10.1038/s41467-022-30559-2 (PMC9156729; doi:10.1038/s41467-022-30559-2)
Supplement: Supplementary file 1 — Supplementary Information [file 41467_2022_30559_MOESM1_ESM.pdf]

Supplementary information for

Neddylation inhibition induces glutamine uptake and metabolism by targeting CRL3^{SPOP} E3 ligase in cancer cells

Qiyin Zhou,[1,2,3,4,5] Wenyu Lin,[2] Chaoqun Wang,[2,6] Fei Sun,[7] Siwei Ju,[8] Qian Chen,[1,4] Yi Wang,[7] Yongxia Chen,[8] Haomin Li,[9] Linbo Wang,[8] Zeping Hu,[7] Hongchuan Jin,[2,5] Xian Wang, [2,3,5,11] and Yi Sun[1,4,5,10,11,*]

[1]Cancer Institute, the Second Affiliated Hospital, Zhejiang University School of Medicine, Hangzhou 310009, China

[2]Key Lab of Biotherapy in Zhejiang, Sir Run Run Shaw Hospital, Zhejiang University School of Medicine, Hangzhou 310016, China

[3]Department of Medical Oncology, Sir Run Run Shaw Hospital, Zhejiang University School of Medicine, Hangzhou 310016, China

[4]Institute of Translational Medicine, Zhejiang University School of Medicine, Hangzhou 310029, China

[5]Cancer Center, Zhejiang University, Hangzhou 310058, China

[6]Department of Pathology, Affiliated Dongyang Hospital of Wenzhou Medical University, Dongyang 322100, China

[7]School of Pharmaceutical Sciences, Tsinghua-Peking Joint Center for Life Sciences, Beijing Frontier Research Center for Biological Structure, Tsinghua University, Beijing 100084, China

[8]Department of Surgical Oncology, Sir Run Run Shaw Hospital, Zhejiang University, Hangzhou 310016, China

[9]Children's Hospital, Zhejiang University School of Medicine, National Clinical Research Center for Child Health, Hangzhou 310052, China

[10]Research Center for Life Science and Human Health, Binjiang Institute of Zhejiang University, Hangzhou 310053, China

[11]These authors jointly supervised this work

Yi Sun at yisun@zju.edu.cn and Xian Wang at wangx118@zju.edu.cn

*Lead contact

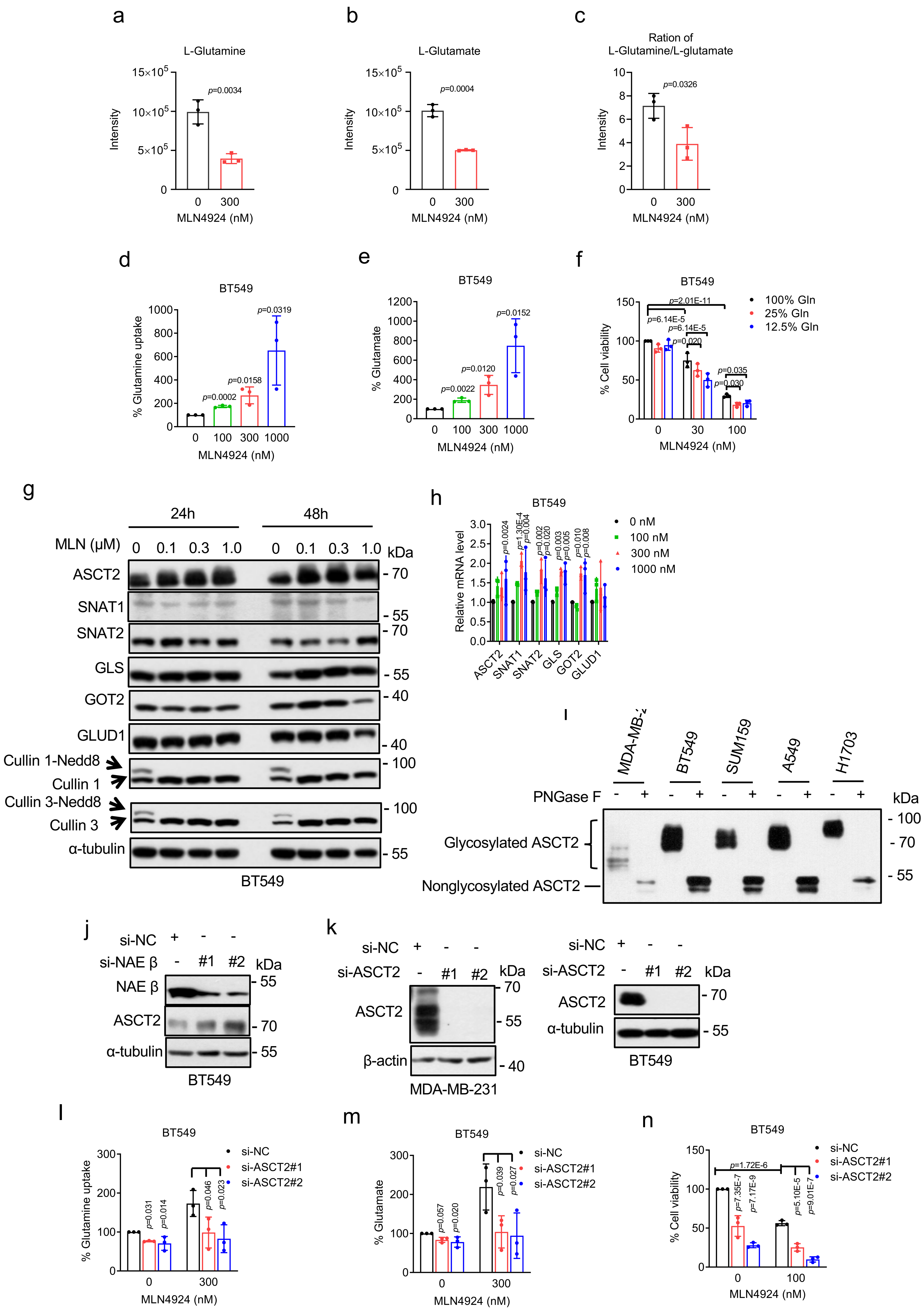
a
L-Glutamine
Intensity
15×10^5
10×10^5
5×10^5
0
p=0.0034
0
300
MLN4924 (nM)
b
L-Glutamate
Intensity
p=0.0004
MLN4924 (nM)
c
Ration of
L-Glutamine/L-glutamate
Intensity
p=0.0326
MLN4924 (nM)
d
BT549
% Glutamine uptake
p=0.0002
p=0.0158
p=0.0319
0
100
300
1000
MLN4924 (nM)
e
BT549
% Glutamate
p=0.0022
p=0.0120
p=0.0152
MLN4924 (nM)
f
BT549
% Cell viability
p=2.01E-11
p=6.14E-5
p=6.14E-5
p=0.020
p=0.035
p=0.030
100% Gln
25% Gln
12.5% Gln
0
30
100
MLN4924 (nM)
g
24h
48h
MLN (μM)
0
0.1
0.3
1.0
kDa
ASCT2
SNAT1
SNAT2
GLS
GOT2
GLUD1
Cullin 1-Nedd8
Cullin 1
Cullin 3-Nedd8
Cullin 3
α-tubulin
BT549
h
BT549
Relative mRNA level
p=0.0024
p=1.30E-4
p=0.004
p=0.002
p=0.020
p=0.003
p=0.005
p=0.010
p=0.008
0 nM
100 nM
300 nM
1000 nM
ASCT2
SNAT1
SNAT2
GLS
GOT2
GLUD1
i
BT549
SUM159
A549
H1703
PNGase F
kDa
Glycosylated ASCT2
Nonglycosylated ASCT2
j
si-NC
si-NAE β
#1
#2
kDa
NAE β
ASCT2
α-tubulin
BT549
k
si-NC
si-ASCT2
#1
#2
kDa
ASCT2
β-actin
MDA-MB-231
ASCT2
α-tubulin
BT549
l
BT549
% Glutamine uptake
p=0.031
p=0.014
p=0.046
p=0.023
si-NC
si-ASCT2#1
si-ASCT2#2
0
300
MLN4924 (nM)
m
BT549
% Glutamate
p=0.057
p=0.020
p=0.039
p=0.027
si-NC
si-ASCT2#1
si-ASCT2#2
MLN4924 (nM)
n
BT549
% Cell viability
p=1.72E-6
p=7.35E-7
p=7.17E-9
p=5.10E-5
p=9.01E-7
si-NC
si-ASCT2#1
si-ASCT2#2
0
100
MLN4924 (nM)

Supplementary Fig. 1. MLN4924 promotes glutamine metabolism through ASCT2.
(**a-c**) MDA-MB-231 cells were treated without or with 300 nM MLN4924 for 24h and subjected to untargeted metabolic profiling analysis, box plots of metabolites levels from cell extracts (glutamine and glutamate) (**a, b**) and culture supernatant (**c**). n=3 with mean ± SD.
(**d, e**) BT549 cells were treated with indicated concentrations of MLN4924 for 24h, and then the culture media were collected to analyze glutamine uptake (**d**) and glutamate production (**e**) (mean ± SD, n = 3).
(**f**) BT549 cells were treated with indicated concentrations of MLN4924 under variable glutamine levels for 48h, and cell viability was detected by trypan blue exclusion assay (mean ± SD, n = 3).
(**g**) BT549 cells were treated with various concentrations of MLN4924 for 24h or 48h and analyzed by immunoblotting.
(**h**) BT549 cells were treated with various concentrations of MLN4924 for 24h, followed by qRT-PCR analysis (mean ± SD, n=3).
(**i**) Cell lysates from various cancer cell lines were treated without or with N-glycosidase F, and then subjected to immunoblotting to detect ASCT2.
(**j**) BT549 cells were transfected with siRNAs targeting NAEβ or scramble siRNA for 48h, followed by immunoblotting.
(**k**) MDA-MB-231 and BT549 cells were transfected with indicated siRNAs against ASCT2 for 48h, followed by immunoblotting.
(**l, m**) BT549 cells were transfected with indicated siRNAs against ASCT2 for 24h, then treated with 300 nM MLN4934, followed by glutamine uptake (**l**) and glutamate production (**m**) detection after 24h (mean ± SD, n=3).
(**n**) BT549 cells were transfected with scramble siRNA (si-NC) or siRNA targeting ASCT2 (si-ASCT2) and with DMSO control or 100 nM MLN4924, followed by trypan blue exclusion assay for cell viability after 48h (mean ± SD, n=3).
Two-tailed, unpaired *t* test for **a**, **b**, **c**, **d**, **e**. O-way ANOVA/LSD test for **f**, **h**, **l**, **m**, **n**. Source data are provided as a Source Data file.

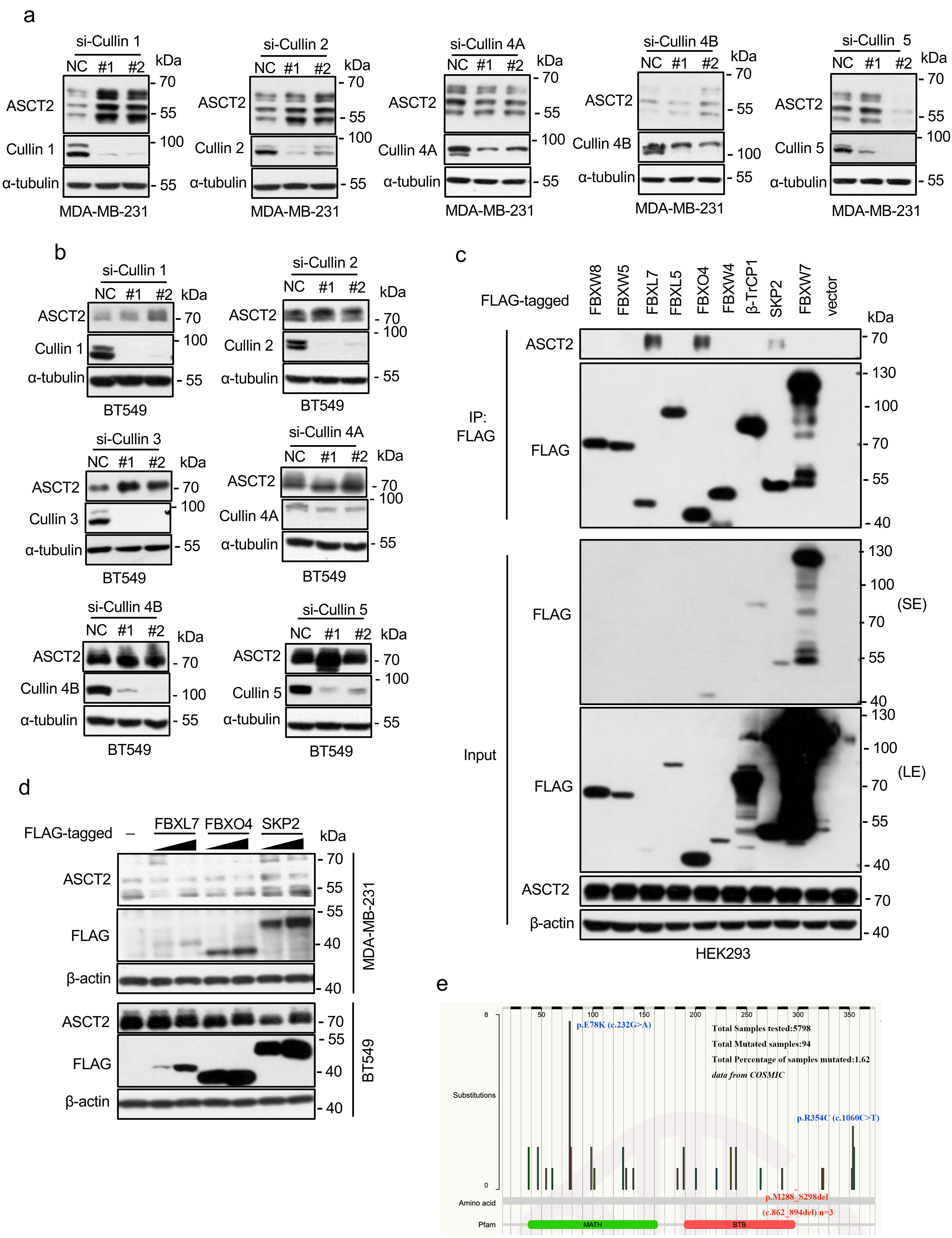

a
si-Cullin 1
NC #1 #2
kDa
ASCT2
Cullin 1
α-tubulin
MDA-MB-231
si-Cullin 2
Cullin 2
si-Cullin 4A
Cullin 4A
si-Cullin 4B
Cullin 4B
si-Cullin 5
Cullin 5
70
55
100
b
si-Cullin 3
Cullin 3
BT549
c
FLAG-tagged
FBXW8
FBXW5
FBXL7
FBXL5
FBXO4
FBXW4
β-TrCP1
SKP2
FBXW7
vector
IP: FLAG
FLAG
Input
(SE)
(LE)
β-actin
HEK293
130
40
d
FBXL7
FBXO4
SKP2
e
p.E78K (c.232G>A)
Total Samples tested:5798
Total Mutated samples:94
Total Percentage of samples mutated:1.62
data from COSMIC
p.R354C (c.1060C>T)
Substitutions
Amino acid
Pfam
MATH
BTB
p.M288_S298del
(c.862_894del) n=3

Supplementary Fig. 2. SPOP interacts with ASCT2.
(**a**) MDA-MB-231 cells were transfected with indicated siRNAs targeting Cullin 1, Cullin 2, Cullin 4A, Cullin 4B, and Cullin 5, respectively, for 48h, and then subjected to immunoblotting.
(**b**) BT549 cells were transfected with siRNAs targeting Cullin 1, Cullin 2, Cullin 3, Cullin 4A, Cullin 4B, and Cullin 5, respectively, for 48h, and then subjected to immunoblotting.
(**c**) HEK293 cells were transfected plasmids for 48h, immunoprecipitated with FLAG-agarose beads and analyzed by immunoblotting.
(**d**) MDA-MB-231 and BT549 cells were transfected with increasing amounts of plasmids for 48h, and analyzed by immunoblotting.
(**e**) SPOP mutations detected in breast cancer tissues.
Source data are provided as a Source Data file.

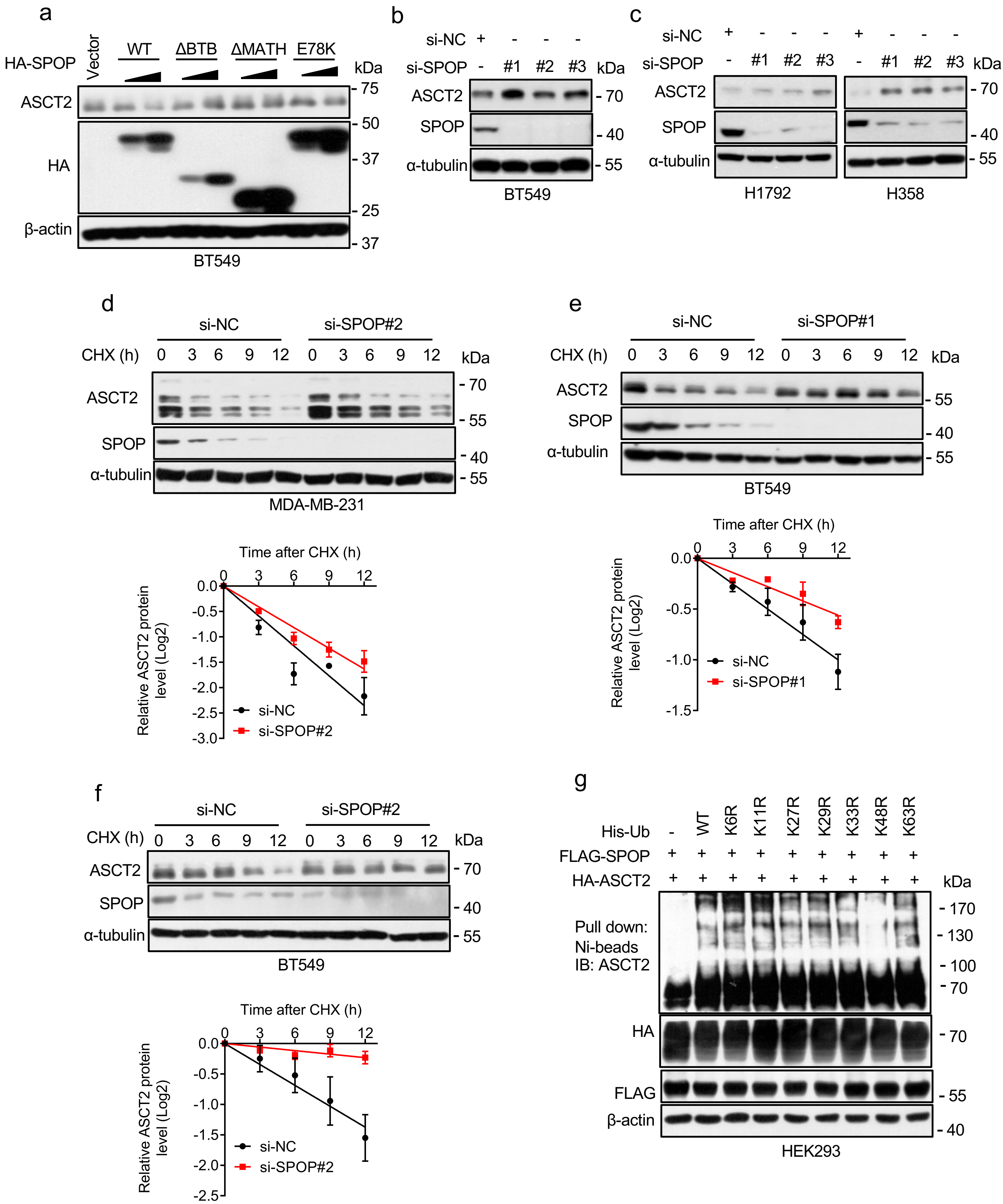
a
HA-SPOP
Vector
WT
ΔBTB
ΔMATH
E78K
kDa
ASCT2
HA
β-actin
75
50
37
25
37
BT549
b
si-NC
si-SPOP
#1
#2
#3
ASCT2
SPOP
α-tubulin
70
40
55
BT549
c
si-NC
si-SPOP
ASCT2
SPOP
α-tubulin
H1792
H358
d
si-NC
si-SPOP#2
CHX (h)
0 3 6 9 12
ASCT2
SPOP
α-tubulin
MDA-MB-231
Time after CHX (h)
Relative ASCT2 protein level (Log2)
si-NC
si-SPOP#2
e
si-NC
si-SPOP#1
CHX (h)
BT549
Time after CHX (h)
Relative ASCT2 protein level (Log2)
si-NC
si-SPOP#1
f
si-NC
si-SPOP#2
CHX (h)
BT549
Time after CHX (h)
Relative ASCT2 protein level (Log2)
si-NC
si-SPOP#2
g
His-Ub
WT
K6R
K11R
K27R
K29R
K33R
K48R
K63R
FLAG-SPOP
HA-ASCT2
Pull down: Ni-beads IB: ASCT2
HA
FLAG
β-actin
170
130
100
70
55
40
HEK293

Supplementary Fig. 3. ASCT2 stability is negatively regulated by SPOP.
(**a**) BT549 cells were transfected with increasing amounts of HA-SPOP and mutants for 48h and analyzed by immunoblotting.
(**b, c**) BT549 (B), H1792, and H358 (**c**) cells were transfected siRNA targeting SPOP or scramble siRNA for 48h, followed by immunoblotting.
(**d**) MDA-MB-231 cells were transfected with siRNA targeting SPOP (si-SPOP#2) or scramble control siRNA for 48h, then incubated with cycloheximide (CHX). Cells were harvested at indicated periods of time for immunoblotting. The band density of ASCT2 was quantified using ImageJ software and normalized to α-tubulin (mean ± SD, n=3).
(**e, f**) BT549 cells were transfected with siRNA targeting SPOP (si-SPOP#1 or si-SPOP#2) or scramble siRNA for 48h, then incubated with cycloheximide (CHX). Cells were harvested at indicated periods of time for immunoblotting. The band density of ASCT2 was quantified using ImageJ software and normalized to α-tubulin (mean ± SD, n=3), respectively.
(**g**) HEK293 cells were transfected with indicated ubiquitin plasmids, lysed under denaturing conditions, followed by Ni-beads pulldown and immunoblotting for ASCT2.
Source data are provided as a Source Data file.

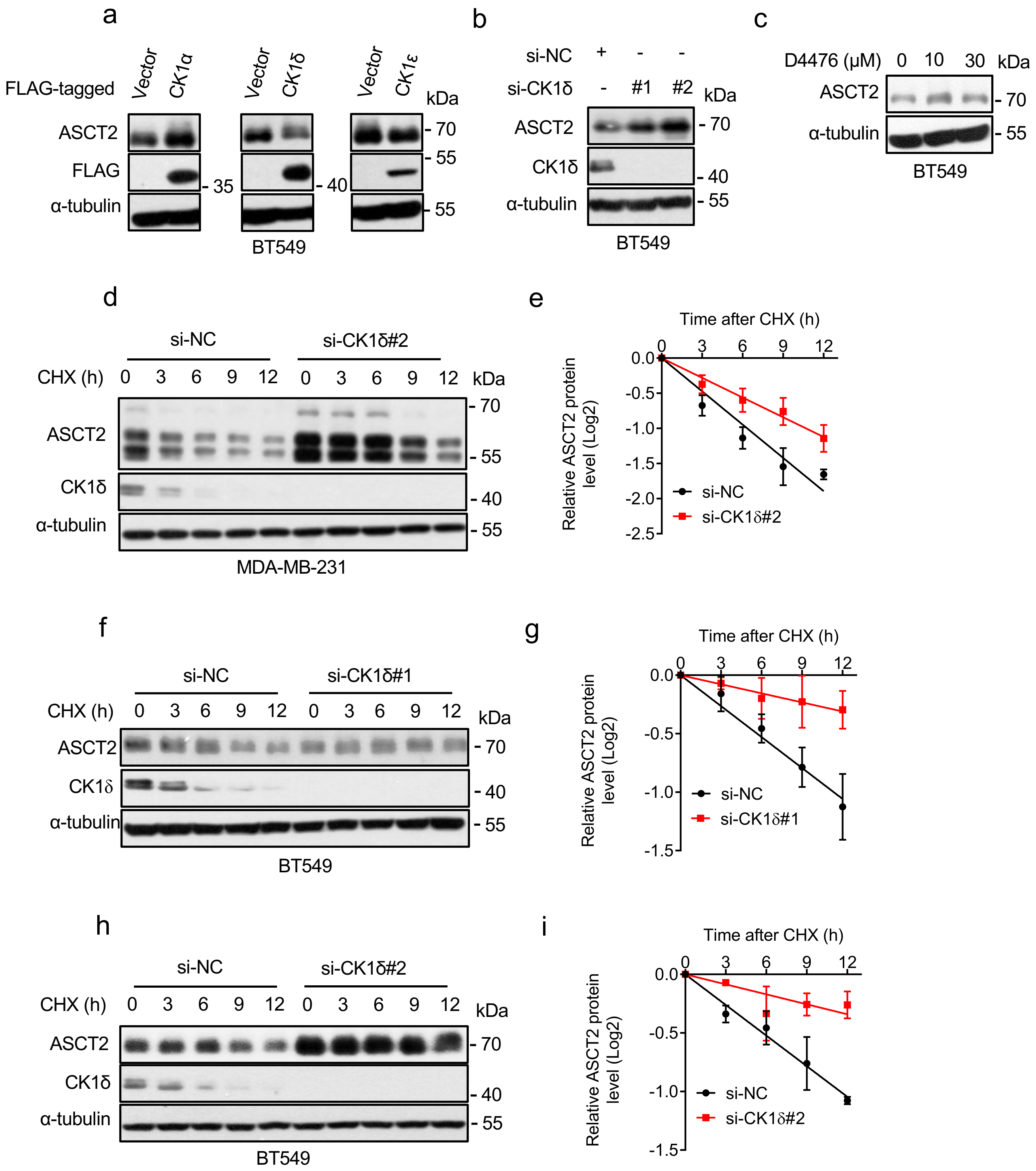

a
FLAG-tagged
Vector
CK1α
Vector
CK1δ
Vector
CK1ε
kDa
ASCT2
FLAG
α-tubulin
- 35
- 40
- 70
- 55
- 55
BT549
b
si-NC + - -
si-CK1δ - #1 #2
kDa
ASCT2
CK1δ
α-tubulin
- 70
- 40
- 55
BT549
c
D4476 (μM) 0 10 30 kDa
ASCT2
α-tubulin
- 70
- 55
BT549
d
si-NC
si-CK1δ#2
CHX (h) 0 3 6 9 12 0 3 6 9 12 kDa
ASCT2
CK1δ
α-tubulin
- 70
- 55
- 40
- 55
MDA-MB-231
e
Time after CHX (h)
Relative ASCT2 protein level (Log2)
si-NC
si-CK1δ#2
f
si-NC
si-CK1δ#1
CHX (h) 0 3 6 9 12 0 3 6 9 12 kDa
ASCT2
CK1δ
α-tubulin
- 70
- 40
- 55
BT549
g
Time after CHX (h)
Relative ASCT2 protein level (Log2)
si-NC
si-CK1δ#1
h
si-NC
si-CK1δ#2
CHX (h) 0 3 6 9 12 0 3 6 9 12 kDa
ASCT2
CK1δ
α-tubulin
- 70
- 40
- 55
BT549
i
Time after CHX (h)
Relative ASCT2 protein level (Log2)
si-NC
si-CK1δ#2

Supplementary Fig. 4. CK1δ regulates ASCT2 stability.

(**a**) BT549 cells were transfected with indicated CK1 isoforms for 48h, then analyzed by immunoblotting.

(**b**) BT549 cells were transfected siRNA targeting CK1δ for 48h, followed by immunoblotting.

(**c**) BT549 cells were treated with different concentrations of CK1 inhibitor D4476 for 24h, then analyzed by immunoblotting.

(**d**, **e**) MDA-MB-231 cells were transfected with si-NC or siRNA targeting CK1δ (si-CK1δ#2) for 48h, then incubated with CHX for indicated periods of time and analyzed by immunoblotting. (**d**), the band density of ASCT2 was quantified using ImageJ software and normalized to α-tubulin (mean ± SD, n=3) (**e**).

(**f-i**) BT549 cells were transfected with si-NC or siRNA targeting CK1δ (si- CK1δ#1 or si-CK1δ#2) for 48h, then incubated with CHX for indicated periods of time and analyzed by immunoblotting (**f**, **h**) , the band density of ASCT2 was quantified using ImageJ software and normalized to α-tubulin (mean ± SD, n=3) (**g**, **i**), respectively.

Source data are provided as a Source Data file.

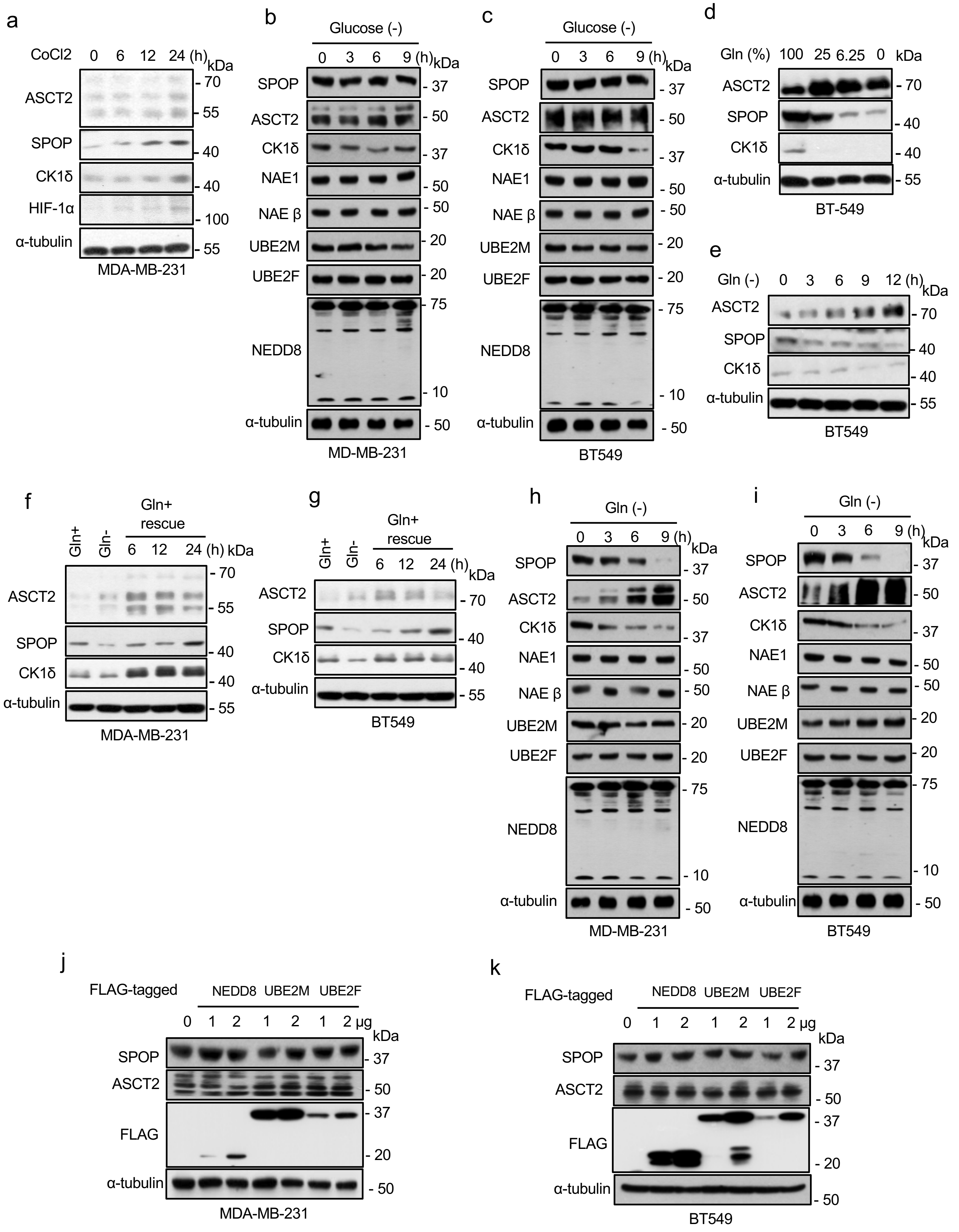
a
CoCl2 0 6 12 24 (h) kDa
ASCT2 - 70 - 55
SPOP - 40
CK1δ - 40
HIF-1α - 100
α-tubulin - 55
MDA-MB-231
b
Glucose (-)
0 3 6 9 (h) kDa
SPOP - 37
ASCT2 - 50
CK1δ - 37
NAE1 - 50
NAE β - 50
UBE2M - 20
UBE2F - 20
NEDD8 - 75 - 10
α-tubulin - 50
MD-MB-231
c
Glucose (-)
0 3 6 9 (h) kDa
SPOP - 37
ASCT2 - 50
CK1δ - 37
NAE1 - 50
NAE β - 50
UBE2M - 20
UBE2F - 20
NEDD8 - 75 - 10
α-tubulin - 50
BT549
d
Gln (%) 100 25 6.25 0 kDa
ASCT2 - 70
SPOP - 40
CK1δ - 40
α-tubulin - 55
BT-549
e
Gln (-) 0 3 6 9 12 (h) kDa
ASCT2 - 70
SPOP - 40
CK1δ - 40
α-tubulin - 55
BT549
f
Gln+ Gln- Gln+ rescue
6 12 24 (h) kDa
ASCT2 - 70 - 55
SPOP - 40
CK1δ - 40
α-tubulin - 55
MDA-MB-231
g
Gln+ Gln- Gln+ rescue
6 12 24 (h) kDa
ASCT2 - 70
SPOP - 40
CK1δ - 40
α-tubulin - 55
BT549
h
Gln (-)
0 3 6 9 (h) kDa
SPOP - 37
ASCT2 - 50
CK1δ - 37
NAE1 - 50
NAE β - 50
UBE2M - 20
UBE2F - 20
NEDD8 - 75 - 10
α-tubulin - 50
MD-MB-231
i
Gln (-)
0 3 6 9 (h) kDa
SPOP - 37
ASCT2 - 50
CK1δ - 37
NAE1 - 50
NAE β - 50
UBE2M - 20
UBE2F - 20
NEDD8 - 75 - 10
α-tubulin - 50
BT549
j
FLAG-tagged
NEDD8 UBE2M UBE2F
0 1 2 1 2 1 2 μg kDa
SPOP - 37
ASCT2 - 50
FLAG - 37 - 20
α-tubulin - 50
MDA-MB-231
k
FLAG-tagged
NEDD8 UBE2M UBE2F
0 1 2 1 2 1 2 μg kDa
SPOP - 37
ASCT2 - 50
FLAG - 37 - 20
α-tubulin - 50
BT549

Supplementary Fig. 5. Reponses of various proteins to stressed conditions.
(**a**) MDA-MB-231 cells were treated with 50 μM $CoCl_2$ for indicated time, and then subjected to immunoblotting.
(**b**, **c**) MDA-MB-231(**b**) and BT549 (**c**) cells were cultured with glucose-free medium for indicated time, and then subjected to immunoblotting.
(**d**) BT549 cells were cultured with indicated doses of glutamine for 24h, and then subjected to immunoblotting.
(**e**) BT549 cells were cultured with glutamine-free medium for indicated time, and then subjected to immunoblotting.
(**f, g**) MDA-MB-231 (**f**) and BT549 (**g**) cells were cultured with glutamine-free medium for 12h, followed by glutamine addition for indicated time points and analyzed by immunoblotting.
(**h, i**) MDA-MB-231 (**h**) and BT549 (**i**) cells were cultured with glutamine-free medium for indicated time, and then subjected to immunoblotting.
(**j, k**) MDA-MB-231(**j**) and BT549 (**k**) cells were transfected with indicated plasmids for 48 h, and then subjected to immunoblotting.
Source data are provided as a Source Data file.

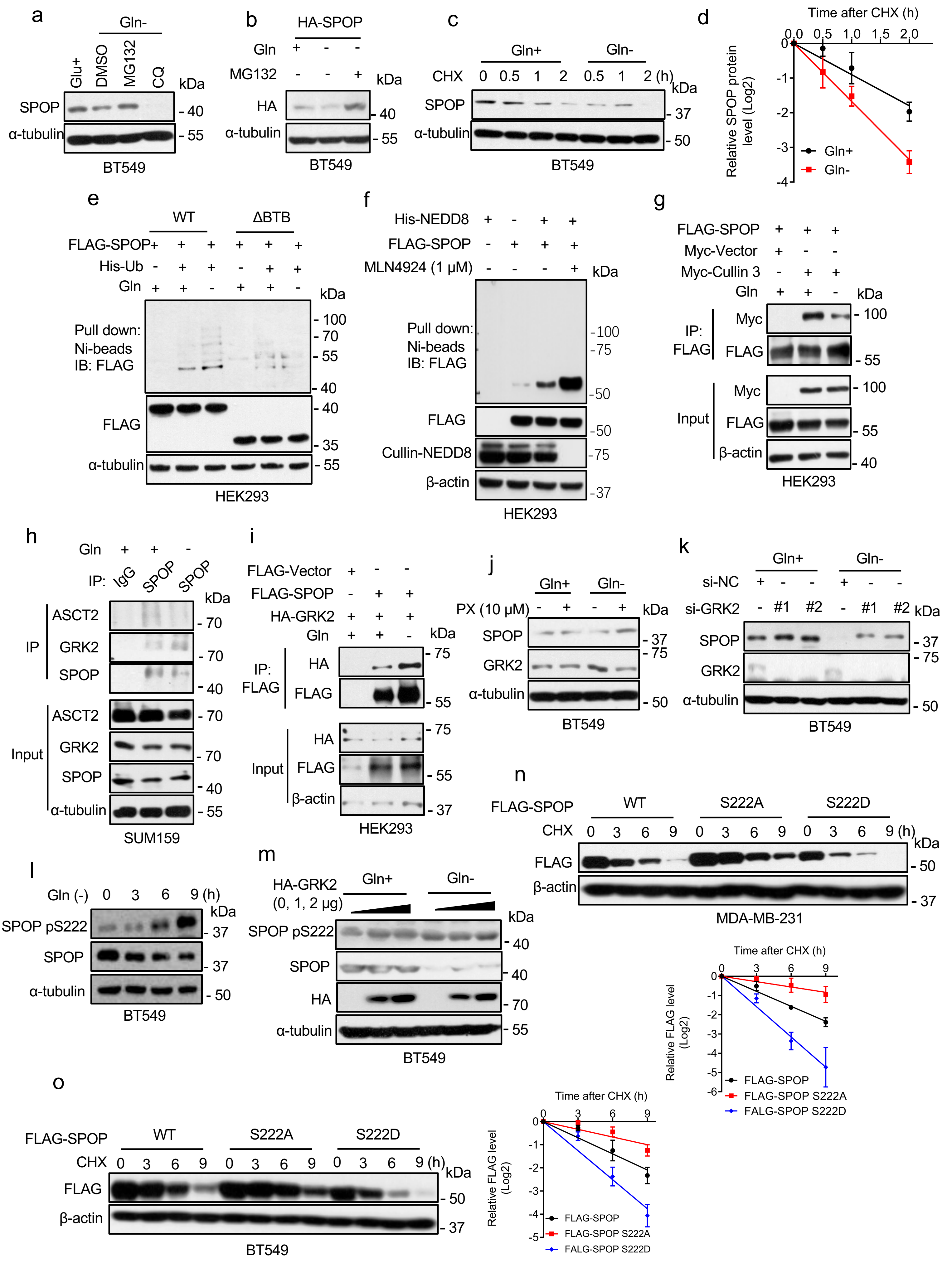

a
Gln-
Glu+
DMSO
MG132
CQ
kDa
SPOP
α-tubulin
40
55
BT549
b
HA-SPOP
Gln
MG132
kDa
HA
α-tubulin
40
55
BT549
c
Gln+
Gln-
CHX 0 0.5 1 2 0.5 1 2 (h)
kDa
SPOP
α-tubulin
37
50
BT549
d
Time after CHX (h)
Relative SPOP protein level (Log2)
Gln+
Gln-
e
WT
ΔBTB
FLAG-SPOP
His-Ub
Gln
kDa
Pull down: Ni-beads IB: FLAG
FLAG
α-tubulin
HEK293
f
His-NEDD8
FLAG-SPOP
MLN4924 (1 μM)
kDa
Pull down: Ni-beads IB: FLAG
FLAG
Cullin-NEDD8
β-actin
HEK293
g
FLAG-SPOP
Myc-Vector
Myc-Cullin 3
Gln
kDa
IP: FLAG
Myc
FLAG
Input
Myc
FLAG
β-actin
HEK293
h
Gln
IP: IgG SPOP SPOP
kDa
IP
ASCT2
GRK2
SPOP
Input
ASCT2
GRK2
SPOP
α-tubulin
SUM159
i
FLAG-Vector
FLAG-SPOP
HA-GRK2
Gln
kDa
IP: FLAG
HA
FLAG
Input
HA
FLAG
β-actin
HEK293
j
Gln+
Gln-
PX (10 μM)
kDa
SPOP
GRK2
α-tubulin
BT549
k
Gln+
Gln-
si-NC
si-GRK2 #1 #2 #1 #2
kDa
SPOP
GRK2
α-tubulin
BT549
l
Gln (-) 0 3 6 9 (h)
kDa
SPOP pS222
SPOP
α-tubulin
BT549
m
HA-GRK2 (0, 1, 2 μg)
Gln+
Gln-
kDa
SPOP pS222
SPOP
HA
α-tubulin
BT549
n
FLAG-SPOP
WT
S222A
S222D
CHX 0 3 6 9 0 3 6 9 0 3 6 9 (h)
kDa
FLAG
β-actin
MDA-MB-231
Time after CHX (h)
Relative FLAG level (Log2)
FLAG-SPOP
FLAG-SPOP S222A
FALG-SPOP S222D
o
FLAG-SPOP
WT
S222A
S222D
CHX 0 3 6 9 0 3 6 9 0 3 6 9 (h)
kDa
FLAG
β-actin
BT549
Time after CHX (h)
Relative FLAG level (Log2)
FLAG-SPOP
FLAG-SPOP S222A
FALG-SPOP S222D

Supplementary Fig. 6. Glutamine stress regulates SPOP and ASCT2 levels.
(**a**) Immunoblotting of cell lysates from BT549 cells cultured with glutamine-free medium and then treated with MG132 or CQ.
(**b**) Immunoblotting of cell lysates from BT549 cells transfected with indicated plasmid and cultured with glutamine-free medium and MG132 in last 6 h.
(**c, d)** BT549 cells were cultured without or with glutamine-free medium with CHX and harvested at indicated periods for immunoblotting (**c**). The band density of SPOP was quantified and normalized to α-tubulin (mean ± SD, n=3) (**d**).
(**e**) Immunoblotting of the products of *in vivo* ubiquitylation assays from HEK293 cells expressing indicated plasmids and cultured in glutamine-containing or glutamine-free media without and with MG132.
(**f**) Immunoblotting of the products of *in vivo* ubiquitylation assays from HEK293 cells expressing indicated plasmids.
(**g**) Immunoblotting of cell lysates and co-IP samples from HEK293 cells transfected with indicated plasmids and cultured with glutamine-free medium for 12h.
(**h**) Immunoblotting of cell lysates and co-IP samples from SUM159 cells.
(**i**) Immunoblotting of cell lysates and co-IP samples from HEK293 cells transfected with indicated plasmids and cultured with glutamine-free medium for 12 h.
(**j**) Immunoblotting of cell lysates from BT549 cells cultured without or with glutamine-free medium in the presence or absence of GRK2 inhibitor PX for 6 h.
(**k**) Immunoblotting of cell lysates from BT549 cells transfected with indicated siRNAs and cultured without or with glutamine-free medium for 6 h.
(**l**) Immunoblotting of cell lysates from BT549 cells cultured in glutamine-free medium for indicated time points.
(**m**) Immunoblotting of cell lysates from BT549 cells transfected with indicated plasmids and cultured in glutamine-containing or glutamine-free media for indicated periods of time.
(**n**) MDA-MB-231 cells transfected with indicated plasmids and cultured in glutamine-free medium with CHX, harvested at indicated periods of time for Immunoblotting. The band density of FLAG was quantified and normalized to α-tubulin (mean ± SD, n=3).
(**o**) BT549 cells transfected with indicated plasmids and cultured in glutamine-free medium with CHX, harvested at indicated periods of time for Immunoblotting. The band density of FLAG was quantified and normalized to α-tubulin (mean ± SD, n=3).
Source data are provided as a Source Data file.

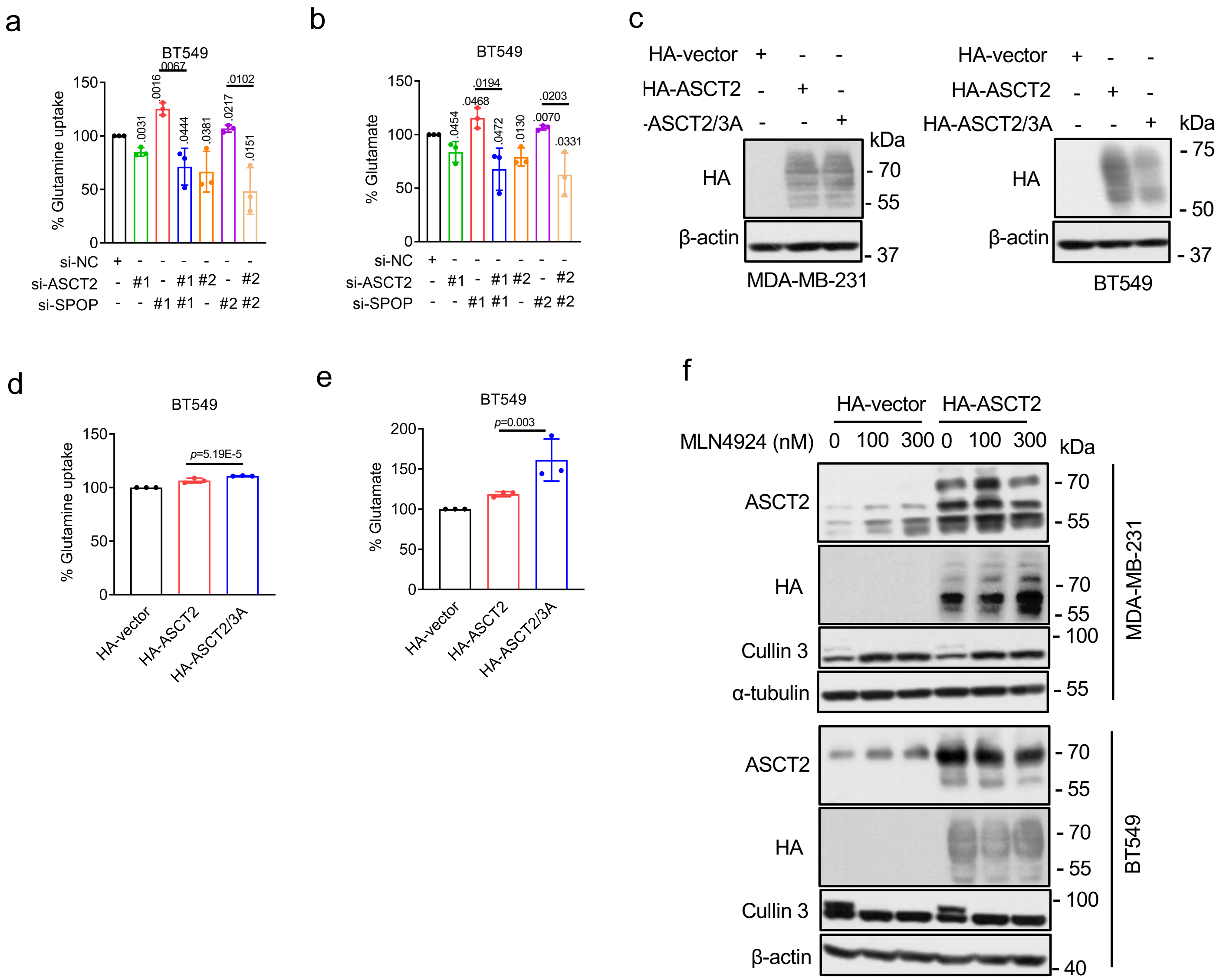

a
BT549
% Glutamine uptake
.0067
.0102
.0016
.0031
.0444
.0381
.0217
.0151
si-NC
si-ASCT2
si-SPOP
b
BT549
% Glutamate
.0194
.0203
.0468
.0454
.0472
.0130
.0070
.0331
si-NC
si-ASCT2
si-SPOP
c
HA-vector
HA-ASCT2
-ASCT2/3A
HA-ASCT2/3A
kDa
HA
β-actin
MDA-MB-231
BT549
d
BT549
% Glutamine uptake
p=5.19E-5
HA-vector
HA-ASCT2
HA-ASCT2/3A
e
BT549
% Glutamate
p=0.003
HA-vector
HA-ASCT2
HA-ASCT2/3A
f
HA-vector
HA-ASCT2
MLN4924 (nM)
kDa
ASCT2
HA
Cullin 3
α-tubulin
β-actin
MDA-MB-231
BT549

Supplementary Fig. 7. Deletion of SPOP stabilizes ASCT2 to promote glutamine metabolism.
(a, b) BT549 cells were transfected with indicated siRNAs for 48h, followed by glutamine uptake (**a**) and glutamate production (**b**) detection (mean ± SD, n=3). **(c)** MDA-MB-231 and BT549 cells were transfected with indicated ASCT2 plasmids for 48h, followed by immunoblotting using indicated antibodies.
(**d, e**) BT549 cells were transfected with wild-type ASCT2 and its mutant ASCT2/3A plasmids for 48h, followed by glutamine uptake (**d**) and glutamate production (**e**) detection (mean ± SD, n=3).
(**f**) MDA-MB-231and BT549 cells were transfected with indicated plasmids, then treated with various concentrations of MLN4924 for 24, and analyzed by immunoblotting.
Two-tailed, unpaired, *t* test for **a**, **b**. O-way ANOVA/LSD test for **d**, **e**. Source data are provided as a Source Data file.

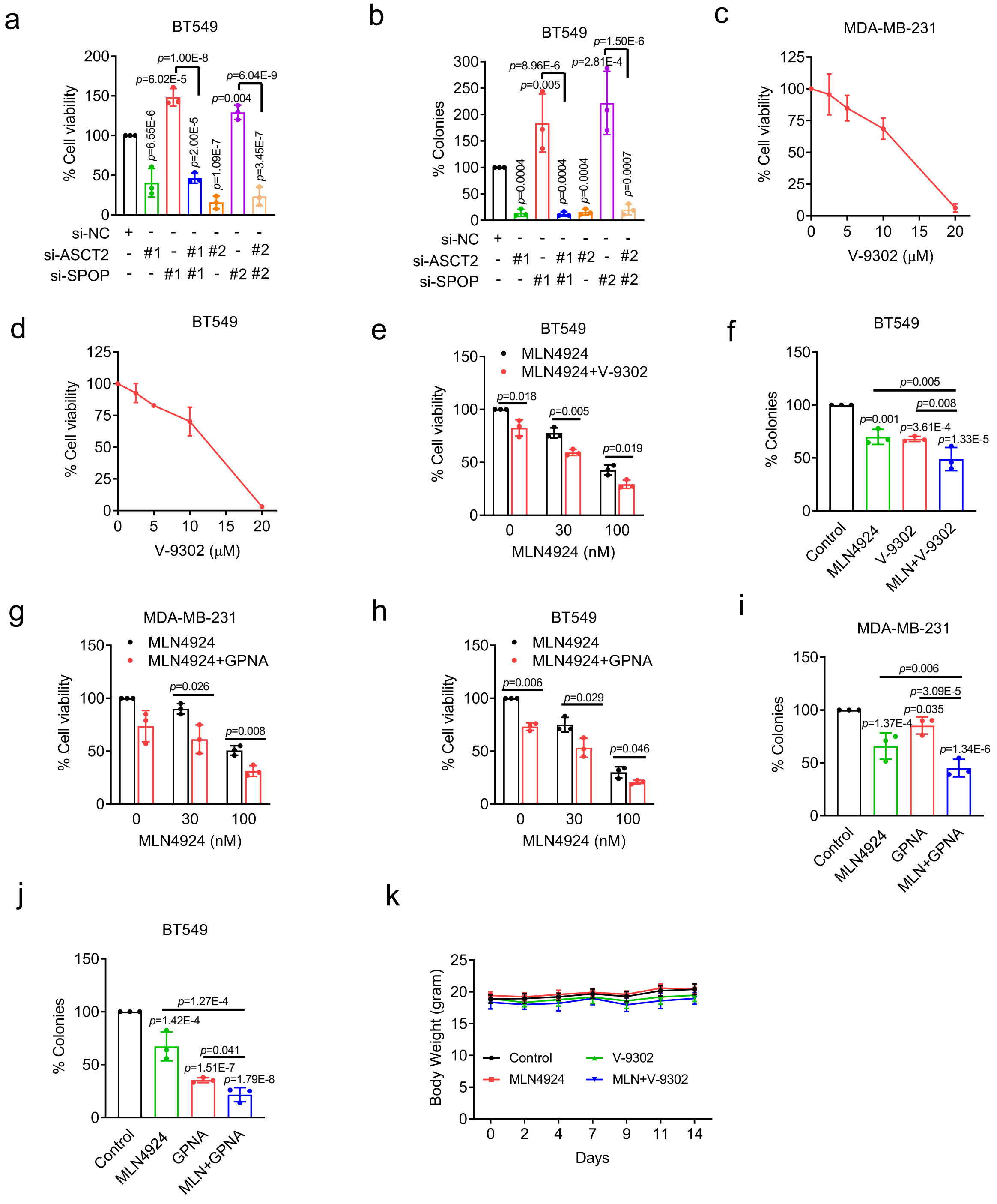
a
BT549
% Cell viability
si-NC
si-ASCT2
si-SPOP
b
BT549
% Colonies
c
MDA-MB-231
% Cell viability
V-9302 (μM)
d
BT549
% Cell viability
V-9302 (μM)
e
BT549
MLN4924
MLN4924+V-9302
% Cell viability
MLN4924 (nM)
f
BT549
% Colonies
Control
MLN4924
V-9302
MLN+V-9302
g
MDA-MB-231
MLN4924
MLN4924+GPNA
% Cell viability
MLN4924 (nM)
h
BT549
MLN4924
MLN4924+GPNA
% Cell viability
MLN4924 (nM)
i
MDA-MB-231
% Colonies
Control
MLN4924
GPNA
MLN+GPNA
j
BT549
% Colonies
Control
MLN4924
GPNA
MLN+GPNA
k
Body Weight (gram)
Control
MLN4924
V-9302
MLN+V-9302
Days

Supplementary Fig. 8. Targeting the SPOP-ASCT2 axis to regulate growth and survival of breast cancer cells

(**a, b**) BT549 cells were transfected with indicated siRNAs and cell viability (**a**) and clonogenic survival (**b**) were measured (mean ± SD, n = 3).

(**c, d**) MDA-Mb231 (**c**) and BT549 (**d**) cells were treated with different concentrations of V-9302 for 72h and cell number was counted through trypan blue exclusion assay (mean ± SD, n=3).

(**e**) BT549 cells were treated with various concentrations of MLN4924 and V-9302 for 72h, then cell number was counted through trypan blue exclusion assay (mean ± SD, n=3).

(**f**) BT549 cells were treated with 5 nM MLN4924, 5 μM V-9302, alone or in combination to measure clonogenic survival (mean ± SD, n = 3).

(**g, h**) MDA-MB-231 (**g**) and BT549 (**h**) cells were treated with various concentrations of MLN4924 and 1 mM GPNA for 72h, then cell number was counted through trypan blue exclusion assay (mean ± SD, n=3).

(**i, j**) MDA-MB-231 (**i**) and BT549 (**j**) cells were treated with 5 nM MLN4924, 1 mM GPNA, alone or in combination to measure clonogenic survival (mean ± SD, n = 3).

(**k**) Body weight of mice carrying MDA-MB-231 xenografts during treatment with MLN4924, V-9302, alone or in combination. For control, V-9302 and MLN4924+V-9302, n=8. For MLN4924, n=7. Mean ± SD.

O-way ANOVA/LSD test for **a**, **b**, **f**, **i**, **j**. Two-tailed, unpaired, *t* test for **e**, **g**, **h**. Source data are provided as a Source Data file.

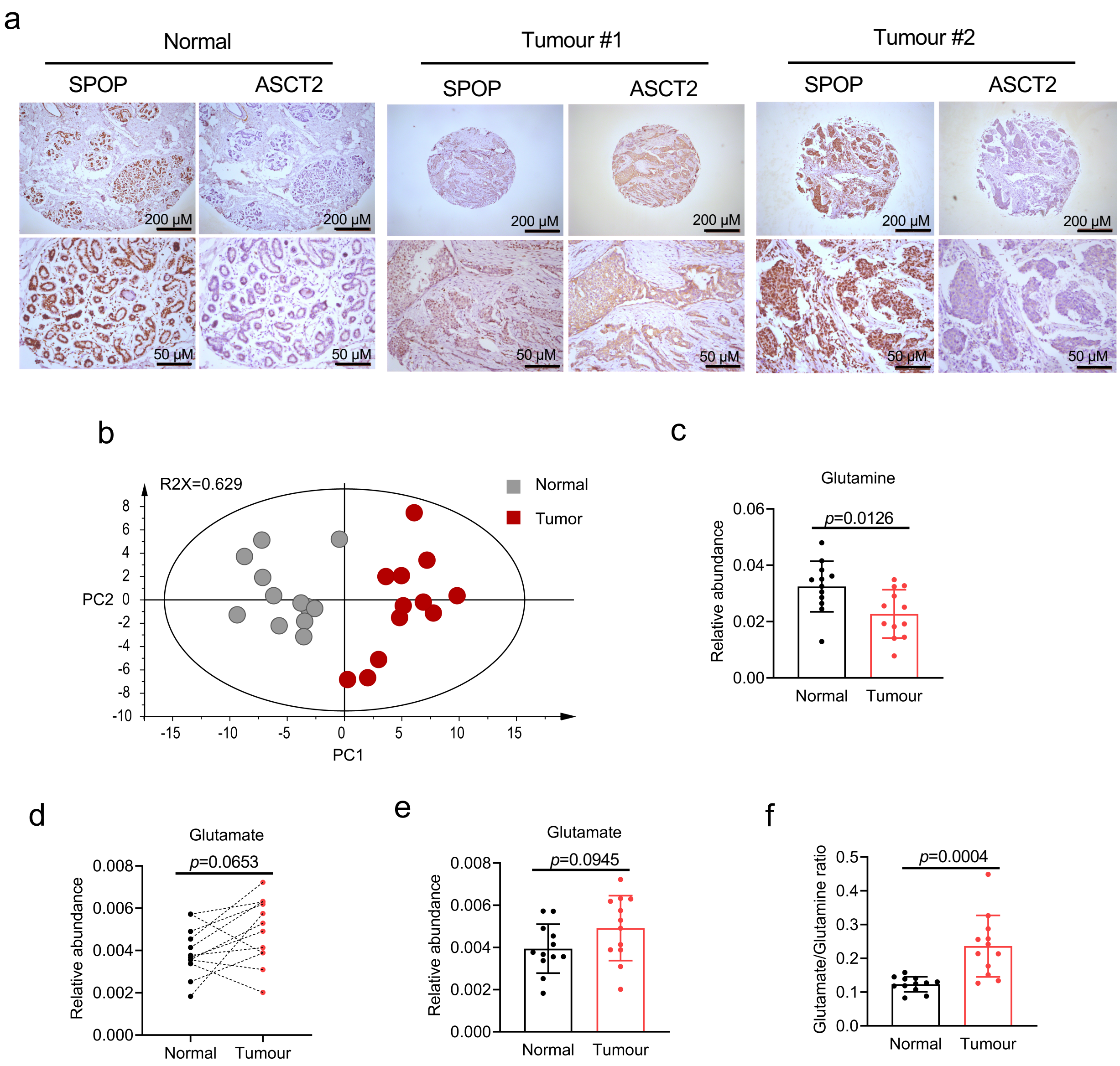

a
Normal
Tumour #1
Tumour #2
SPOP
ASCT2
200 μM
50 μM
b
R2X=0.629
Normal
Tumor
PC2
PC1
c
Glutamine
p=0.0126
Relative abundance
Normal
Tumour
d
Glutamate
p=0.0653
Relative abundance
Normal
Tumour
e
Glutamate
p=0.0945
Relative abundance
Normal
Tumour
f
p=0.0004
Glutamate/Glutamine ratio
Normal
Tumour

Supplementary Fig. 9. Inverse correlation of SPOP and ASCT2, and altered glutamine metabolism in breast cancer tissues.

(**a**) Representative images of SPOP and ASCT2 staining in consecutive breast tissues (normal vs. tumor).

(**b**) Paired human breast normal and tumor tissues were analyzed by targeted metabolomics. Shown is the PCA score plot of tissue metabolites.

(**c-f**) Paired human breast normal and tumor tissues were analyzed by targeted metabolomics, and relative abundance of glutamine (**c**), glutamate (**d, e**), glutamine/glutamate ratio (**f**) were evaluated/compared between total or each individual pair of normal vs. tumor tissues. n=12 with mean ± SD.

Two-tailed, unpaired, *t* test for **c**, **e**, and **f**; Two-tailed, paired t test for **d**. Source data are provided as a Source Data file.
