# Supplementary material for: Neddylation inhibition induces glutamine uptake and metabolism by targeting CRL3SPOP E3 ligase in cancer cells
Source: Nat Commun. 2022 May 31;13:3034. doi: 10.1038/s41467-022-30559-2 (PMC9156729; doi:10.1038/s41467-022-30559-2)
Supplement: Supplementary file 3 — Reporting Summary [file 41467_2022_30559_MOESM3_ESM.pdf]

nature portfolio

Corresponding author(s): Yi Sun
Last updated by author(s): April 25, 2022

Reporting Summary

Nature Portfolio wishes to improve the reproducibility of the work that we publish. This form provides structure for consistency and transparency in reporting. For further information on Nature Portfolio policies, see our Editorial Policies and the Editorial Policy Checklist.

Statistics

For all statistical analyses, confirm that the following items are present in the figure legend, table legend, main text, or Methods section.

n/a	Confirmed	
☐	☒	The exact sample size (n) for each experimental group/condition, given as a discrete number and unit of measurement
☐	☒	A statement on whether measurements were taken from distinct samples or whether the same sample was measured repeatedly
☐	☒	The statistical test(s) used AND whether they are one- or two-sided<br>*Only common tests should be described solely by name; describe more complex techniques in the Methods section.*
☒	☐	A description of all covariates tested
☒	☐	A description of any assumptions or corrections, such as tests of normality and adjustment for multiple comparisons
☐	☒	A full description of the statistical parameters including central tendency (e.g. means) or other basic estimates (e.g. regression coefficient) AND variation (e.g. standard deviation) or associated estimates of uncertainty (e.g. confidence intervals)
☐	☒	For null hypothesis testing, the test statistic (e.g. F, t, r) with confidence intervals, effect sizes, degrees of freedom and P value noted<br>*Give P values as exact values whenever suitable.*
☒	☐	For Bayesian analysis, information on the choice of priors and Markov chain Monte Carlo settings
☒	☐	For hierarchical and complex designs, identification of the appropriate level for tests and full reporting of outcomes
☒	☐	Estimates of effect sizes (e.g. Cohen's d, Pearson's r), indicating how they were calculated

Our web collection on statistics for biologists contains articles on many of the points above.

Software and code

Policy information about availability of computer code

Data collection	Targeted metabolomics data were collected with an AB QTRAP 6500+ triple quadrupole mass spectrometer (SCIEX, Framingham, MA).
Data analysis	ImageJ 1.48v was used for images processing.<br>GraphPad Prism 8.0.2.263 and SPSS 16.0 were used for statistical analysis.<br>SCIEX Multi-Quant 3.0.2 was used for chromatogram review and peak area integration.

For manuscripts utilizing custom algorithms or software that are central to the research but not yet described in published literature, software must be made available to editors and reviewers. We strongly encourage code deposition in a community repository (e.g. GitHub). See the Nature Portfolio guidelines for submitting code & software for further information.

Data

Policy information about availability of data

All manuscripts must include a data availability statement. This statement should provide the following information, where applicable:
- Accession codes, unique identifiers, or web links for publicly available datasets
- A description of any restrictions on data availability
- For clinical datasets or third party data, please ensure that the statement adheres to our policy

All experimental data that support the findings of this study are included as follows: 7 main figures, 9 Supplementary figures, and Source data. Full metabolomics data can be found in the Source Data file. All the data are available from the corresponding author, Yi Sun. Source data are provided with this paper.

Field-specific reporting

Please select the one below that is the best fit for your research. If you are not sure, read the appropriate sections before making your selection.

- [x] Life sciences
- [ ] Behavioural & social sciences
- [ ] Ecological, evolutionary & environmental sciences

For a reference copy of the document with all sections, see nature.com/documents/nr-reporting-summary-flat.pdf

Life sciences study design

All studies must disclose on these points even when the disclosure is negative.

Sample size	No statistical method was used to pre-select the sample size. The sample size was based on the literature that tested the same cell lines or performed the similar assays. Animal numbers for each study type were determined by the investigators on the basis of our previous studies (Zhou et al., JCI Insight.2019, 4(4):e121582. ; Yin et al., PNAS. 2019, 116(41): 20528-20538.).
Data exclusions	There are no data exclusions.
Replication	Experiments were performed 2-3 times. Similar observation was obtained for each replicate. Representative result was shown in the figures.
Randomization	All mice were randomly divided into different groups. Randomization is not applicable to cell culture experiments.
Blinding	The experiments and analysis were performed in a non-blinded fashion because the same investigator was doing group allocation during data collection and analysis. The data generated were analyzed objectively without any preference or bias.

Reporting for specific materials, systems and methods

We require information from authors about some types of materials, experimental systems and methods used in many studies. Here, indicate whether each material, system or method listed is relevant to your study. If you are not sure if a list item applies to your research, read the appropriate section before selecting a response.

Materials & experimental systems

n/a	Involved in the study	
[ ]	[x]	Antibodies
[ ]	[x]	Eukaryotic cell lines
[x]	[ ]	Palaeontology and archaeology
[ ]	[x]	Animals and other organisms
[ ]	[x]	Human research participants
[x]	[ ]	Clinical data
[x]	[ ]	Dual use research of concern

Methods

n/a	Involved in the study	
[x]	[ ]	ChIP-seq
[x]	[ ]	Flow cytometry
[x]	[ ]	MRI-based neuroimaging

Antibodies

Antibodies used	ASCT2 (Cell Signaling Technology, D7C12, 8057, dilution: 1:1000); ASCT2 (Abcam, ab84903, dilution: 1:800); β-actin (Sigma-Aldrich, A5441, dilution: 1:10000); CK1δ (Santa Cruz, sc-55553, dilution: 1:1000); CK1δ (Cell Signaling Technology, 12417S, dilution: 1:1000); CUL-1 (Santa Cruz, sc-11384, dilution: 1:1000); CUL-2 (Abcam, ab166917, dilution: 1:1000); CUL-3 (Cell Signaling Technology, 2759S, dilution: 1:1000); CUL-4A (Cell Signaling Technology, 2699S, dilution: 1:1000); CUL-4B (Proteintech, 12916-1-AP, dilution: 1:1000); CUL-5 (Santa Cruz, sc-13014, dilution: 1:1000); FLAG, clone M2 (Sigma-Aldrich, F1804-500UG, dilution: 1:2000); FLAG M2 affinity gel (Sigma-Aldrich, A2220-5ML); GLUD1 (Abcam, ab89967, dilution: 1:1000); GLS (Abcepta, ap8809b, dilution: 1:1000); GOT2 (ProteinTech, 14800-1-AP, dilution: 1:1000); GRK2 (ProteinTech, 13990-1-AP, dilution: 1:1000); GRK2 (Cell Signaling Technology, 3982, dilution: 1:1000); HA (Sigma, H6908, dilution: 1:2000); Anti-HA High Affinity (3F10) (Roche, 11867423001); HIF-1α (Santa Cruz, sc-53546, dilution: 1:1000); NAEβ (Abcam, ab124728, dilution: 1:1000); NAE1 (Cell Signaling Technology, 14321S, dilution: 1:1000); NEDD8 (Abcam, ab81264, dilution: 1:1000); SNAT1 (Abcam, ab134268, dilution: 1:1000); SNAT2 (Abcam, ab90677, dilution: 1:1000); SPOP (Abcam, ab137537, dilution: 1:1000); SPOP (Affinit, DF12106, dilution: 1:800); SPOP (Santa Cruz, sc-377206, dilution: 1:1000); p-SPOP (Ser222) (dilution: 1:1000); α-tubulin (Sigma, Clone AA13, T8203, dilution: 1:10000); UBE2M (Santa Cruz, sc-390064, dilution: 1:1000); UBE2F (ProteinTech, 26356-1-AP, dilution: 1:1000); Peroxidase AffiniPure Goat Anti-Rabbit IgG (H+L) (Jackson, 11-035-144, dilution: 1:4000); Peroxidase AffiniPure Goat Anti-Mouse IgG (H+L) (Jackson, 115-035-146, dilution: 1:4000); Peroxidase AffiniPure Goat Anti-Rat IgG (H+L) (Jackson, 112-035-143, dilution: 1:4000). A peptide polyclonal Ab against phosphor-SPOP-Ser222 (AILAAR(pS) PVFSA) was generated, followed by two sequential rounds of affinity purification by Youke Biological Technology (Shanghai, China).
Validation	For IHC:<br>1. ASCT2: (Abcam, ab84903, dilution: 1:800), PMID: 30855698<br>2. SPOP: (Affinit, DF12106, dilution: 1:800) http://www.affbiotech.com/goods-15196-DF12106-SPOP_Antibody.html

For Western Blot:
1. ASCT2 (Cell Signaling Technology, D7C12, 8057, dilution: 1:1000), https://www.cellsignal.com/products/primary-antibodies/asct2-d7c12-rabbit-mab/8057?site-search-type=Products&N=4294956287&Ntt=asct2&fromPage=plp
2. β-actin (Sigma-Aldrich, A5441, dilution: 1:10000), https://www.sigmaaldrich.cn/CN/en/product/sigma/a5441
3. CK1δ (Santa Cruz, sc-55553, dilution: 1:1000), https://www.scbt.com/p/casein-kinase-idelta-antibody-c-8?productCanUrl=casein-kinase-idelta-antibody-c-8&_requestid=5824045
4. CK1δ (Cell Signaling Technology, 12417S, dilution: 1:1000), https://www.cellsignal.com/products/primary-antibodies/ck1d-antibody/12417?site-search-type=Products&N=4294956287&Ntt=12417s&fromPage=plp&_requestid=2272770
5. CUL-1 (Santa Cruz, sc-11384, dilution: 1:1000), https://www.scbt.com/p/cul-1-antibody-h-213?requestFrom=search
6. CUL-2 (Abcam, ab166917, dilution: 1:1000), https://www.abcam.com/cullin-2cul-2-antibody-epr31042-ab166917.html
7. CUL-3 (Cell Signaling Technology, 2759S, dilution: 1:1000), https://www.cellsignal.com/products/primary-antibodies/cul3-antibody/2759?site-search-type=Products&N=4294956287&Ntt=2759s&fromPage=plp&_requestid=2273293
8. CUL-4A (Cell Signaling Technology, 2699S, dilution: 1:1000), https://www.cellsignal.com/products/primary-antibodies/cul4a-antibody/2699?site-search-type=Products&N=4294956287&Ntt=2699s&fromPage=plp&_requestid=2273447
9. CUL-4B (Proteintech, 12916-1-AP, dilution: 1:1000), https://www.ptglab.com/products/CUL4B-Antibody-12916-1-AP.htm
10. CUL-5 (Santa Cruz, sc-13014, dilution: 1:1000), https://www.scbt.com/p/cul-5-antibody-h-300?requestFrom=search
11. FLAG, clone M2 (Sigma-Aldrich, F1804-500UG, dilution: 1:2000), https://www.sigmaaldrich.cn/CN/en/product/sigma/f1804
12. FLAG M2 affinity gel (Sigma-Aldrich, A2220-5ML), https://www.sigmaaldrich.cn/CN/en/product/sigma/a2220
13. GLUD1 (Abcam, ab89967, dilution: 1:1000), PMID: 29249655
14. GLS (Abgent, ap8809b, dilution: 1:1000), https://www.abcepta.com/products/AP8809b-GLS-Antibody-C-term
15. GOT2 (ProteinTech, 14800-1-AP, dilution: 1:1000), https://www.ptglab.com/products/GOT2-Antibody-14800-1-AP.htm
16. GRK2 (ProteinTech, 13990-1-AP, dilution: 1:1000), https://www.ptglab.com/products/ADRBK1-Antibody-13990-1-AP.htm
17. GRK2 (Cell Signaling Technology, 3982, dilution: 1:1000), https://www.cellsignal.com/product/productDetail.jsp?productId=3982
18. HA (Sigma, H6908, dilution: 1:2000), https://www.sigmaaldrich.cn/CN/en/product/sigma/h6908
19. Anti-HA High Affinity (3F10) (Roche, 11867423001), https://www.sigmaaldrich.cn/CN/en/product/roche/roahaha
20. HIF-1α (Santa Cruz, sc-53546, dilution: 1:1000), https://www.scbt.com/p/hif-1alpha-antibody-h1alpha-67?requestFrom=search
21. NAEβ (Abcam, ab124728, dilution: 1:1000), https://www.abcam.com/uba3-antibody-milr42872-ab124728.html
22. NAE1 (Cell Signaling Technology, 14321S, dilution: 1:1000), https://www.cellsignal.com/products/primary-antibodies/nae1-appbp1-d9i4z-rabbit-mab/14321?site-search-type=Products&N=4294956287&Ntt=14321s&fromPage=plp&_requestid=2277259
23. NEDD8 (Abcam, ab81264, dilution: 1:1000), https://www.abcam.com/nedd8-antibody-y297-ab81264.html
24. SNAT1 (Abcam, ab134268, dilution: 1:1000), https://www.abcam.com/slc38a1nat2-antibody-n10432-ab134268.html
25. SNAT2 (Abcam, ab90677, dilution: 1:1000), PMID: 30274521
26. SPOP (Abcam, ab137537, dilution: 1:1000), https://www.abcam.com/spop-antibody-ab137537.html
27. SPOP (Santa Cruz, sc-377206, dilution: 1:1000), https://www.scbt.com/p/spop-antibody-b-8?requestFrom=search
28. p-SPOP (Ser222) (dilution: 1:1000), validated by molecular weight in our lab
29. α-tubulin (Sigma, Clone AA13, T8203, dilution: 1:10000), https://www.sigmaaldrich.cn/CN/en/product/sigma/t8203
30. UBE2M (Santa Cruz, sc-390064, dilution: 1:1000), https://www.scbt.com/p/ubc12-antibody-d-4?requestFrom=search
31. UBE2F (ProteinTech, 17056-1-AP, dilution: 1:1000), https://www.ptglab.com/products/UBE2F-Antibody-17056-1-AP.htm
32. Peroxidase AffiniPure Goat Anti-Rabbit IgG (H+L) (Jackson, 11-035-144, dilution: 1:4000), https://www.jacksonimmuno.com/catalog/products/111-035-144
33. Peroxidase AffiniPure Goat Anti-Mouse IgG (H+L) (Jackson, 115-035-146, dilution: 1:1000), https://www.jacksonimmuno.com/catalog/products/115-035-146
34. Peroxidase AffiniPure Goat Anti-Rat IgG (H+L) (Jackson, 112-035-143, dilution: 1:4000), https://www.jacksonimmuno.com/catalog/products/112-035-143

Eukaryotic cell lines

Policy information about cell lines

Cell line source(s)

Human breast cancer cell lines MDA-MB-231 (HTB-26), BT549 (HTB-122), SK-BR-3 (HTB-30), lung cancer cell lines A549 (CCL-185), H1703 (CRL-5889), H1792 (CRL-5895) and H358 (CRL-5807), and embryonic kidney cell line HEK293 (CRL-1573) were obtained from American Type Culture Collection. Human breast cancer cell line SUM159 was a kind gift from Dr. Chenfang Dong, Zhejiang University.

Authentication

All cell lines were authenticated by short tandem repeat (STR) profiling.

Mycoplasma contamination

All cell lines used were negative for mycoplama contamination.

Commonly misidentified lines (See ICLAC register)

No cell lines used in this manuscript were misidentified.

Animals and other organisms

Policy information about studies involving animals; ARRIVE guidelines recommended for reporting animal research

Laboratory animals

Five- to six-week-old BALB/c athymic nude mice (nu/nu, female) were used. These mice were housed in the specific pathogen-free (SPF) environment at a constant temperature (25خ) and a relatively constant humidity with ad libitum access to water and food (40-60%), with 12 h dark/light cycle.

Wild animals	This study did not involve wild animals.
Field-collected samples	No field collected samples were used in the study.
Ethics oversight	All animal studies were approved by and conducted in accordance with the guidelines established by the committee on Use and Care of Animals at the Zhejiang University.

Note that full information on the approval of the study protocol must also be provided in the manuscript.

Human research participants

Policy information about studies involving human research participants

Population characteristics	The patients included in this study were diagnosed with breast cancer and underwent surgical resection at Sir Run Run Shaw Hospital, Zhejiang University School of Medicine. For Immunohistochemistry (IHC) staining of human breast cancer tissues, 314 cases were female, with ages ranging from 24 to 75. For Targeted metabolomics analysis, 12 cases were female, with ages ranging from 38 to 74.
Recruitment	The patients included in this study were diagnosed with breast cancer and underwent surgical resection at Sir Run Run Shaw Hospital, Zhejiang University School of Medicine. All participants provided written and informed consent. There was no apparent biases in the recruitment of the patients.
Ethics oversight	The study was approved by the ethical committee of Sir Run Run Shaw Hospital, Zhejiang University School of Medicine.

Note that full information on the approval of the study protocol must also be provided in the manuscript.
